# Physiological and transcriptome analyses reveal the response of *Ammopiptanthus mongolicus* to extreme seasonal temperatures in a cold plateau desert ecosystem

**DOI:** 10.1038/s41598-022-14402-8

**Published:** 2022-06-23

**Authors:** Zimeng Yang, Yiying Liu, Hang Han, Xinyu Zhao, Siyu Chen, Guofang Li, Sha Shi, Jinchao Feng

**Affiliations:** 1 Key Laboratory of Ecology and Environment in Minority Areas, (Minzu University of China), National Ethnic Affairs Commission, Beijing, 100081 China; 2 College of Life and Environmental Sciences, Minzu University of China, Beijing, 100081 China

**Keywords:** Ecophysiology, Molecular ecology

## Abstract

*Ammopiptanthus mongolicus* is the only evergreen broad-leaved shrub present in arid areas of Northwest China and plays an important role in maintaining the stability of the local desert ecosystem. It can survive under extreme temperatures (e.g., extreme low temperature: − 24.8 °C and extreme high temperature: 37.7 °C). To understand the gene expression-physiological regulation network of *A. mongolicus* in extreme temperature environments, we monitored the changes in gene expression and photosynthetic traits of the leaves. The results showed that at low temperatures, the net photosynthetic rates (A), Fv'/Fm' and electron transport rate (ETR) decreased, the Fv/Fm ratio was only 0.32, and the proportion of nonregulatory heat dissipation Y(NO) increased. Based on a KEGG analysis of the differentially expressed genes, 15 significantly enriched KEGG pathways were identified, which were mainly related to circadian rhythm, photosynthesis, lipid metabolism, carbohydrate metabolism, plant hormones and other life activities. At high temperatures, the A value increased, and the proportion of regulatory energy dissipation Y(NPQ) increased. The KEGG analysis identified 24 significantly enriched KEGG pathways, which are mainly related to circadian rhythm, carbon sequestration of photosynthesis, carotenoid biosynthesis, secondary metabolites, cofactors and vitamin metabolism. In general, at the expense of photosynthesis, *A. mongolicus* can ensure the survival of leaves by increasing Y(NO) levels, regulating the circadian rhythm, increasing the synthesis of unsaturated fatty acids and changing the role of plant hormones. Under high-temperature stress, a high photosynthetic capacity was maintained by adjusting the stomatal conductance (g_sw_), increasing Y(NPQ), consuming excess light energy, continuously assembling and maintaining PSII, and changing the production of antioxidants.

## Introduction

*Ammopiptanthus mongolicus* is a leguminous shrub that is mainly distributed in Inner Mongolia, Ningxia, Gansu and other provinces in China. *A. mongolicus* is the only evergreen broad-leaved shrub present in the arid areas of Northwest China and is one of the dominant and constructive species of in the desert vegetation in this region and plays an important role in maintaining the stability of the local desert ecosystem^1^. At the same time, as an ancient relic species of the third season, *A. mongolicus* is very resistant to cold and drought and can grow under extreme temperatures. It is an ideal material for studying the stress resistance and adaptability of desert plants.

Studies have shown that evergreens retain their foliage amid low-temperature environmental conditions. The enhancement of foliar resistance to environmental stressors typically occurs at the expense of carbon assimilation^2,3^. To adapt to various extreme conditions in desert ecosystems, *A. mongolicus* has evolved a variety of physiological adaptation mechanisms, which are highlighted in the leaves, an important organ of photosynthesis. In spring and summer, *A. mongolicus* absorbs carbon dioxide to the maximum extent possible to maintain a high photosynthetic capacity. Under winter conditions, photooxidative damage to leaf tissues is averted by increasing the level of dark-sustained thermal energy dissipation. However, studies on the gene expression regulation in response to the stress environment of *A. mongolicus* have mainly taken place in the laboratory, and artificially cultured *A. mongolicus* seedlings were treated with a single artificial stress^4–6^. These experiments are not adequate to explain the physiological response mechanisms of *A. mongolicus* under natural conditions and are not convincing as a scientific basis for vegetation protection of desert ecosystems. Therefore, the natural *A. mongolicus* population in West Ordos, Inner Mongolia, China, was selected as the research object. We examined the "temperature" changes, which are a key ecological factor in the field, determined the physiological and ecological indices of leaves under naturally occurring extreme temperature conditions, and collected leaf samples for transcriptome analysis. By combining transcriptional regulation with physiological regulation, this paper explains the adaptation mechanism of *A. mongolicus* to extreme seasonal desert temperatures at multiple levels to provide feasible suggestions for protecting natural desert ecosystems.

## Results

### Physiological characteristics of A. mongolicus. under different temperatures

**Figure 1:**
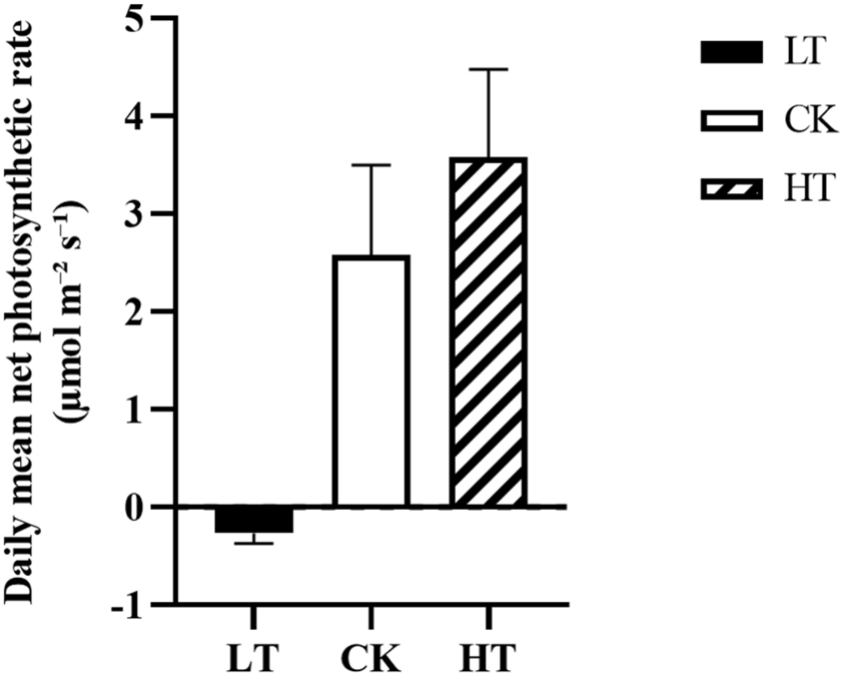
Daily mean net photosynthetic rates (**A**). The columns represent the mean ± SE (n = 5). The letters above the columns indicate significant differences among groups. LT: low temperature treatment group, CK: control group, and HT: high temperature treatment group.

The daily average value of the net photosynthetic rate (A) in the LT treatment group was negative, which indicated that daytime photosynthesis was lower than respiration. The plant sacrifices of dry matter accumulation ensure the survival of leaves, which means that the leaves of *A. mongolicus* stop growing. The daily average A value of the high-temperature treatment group (HT) increased by 39% compared with the control group (CK), which indicated that *A. mongolicus* was well adapted to high-temperature stress (Fig. 1).

**Figure 2:**
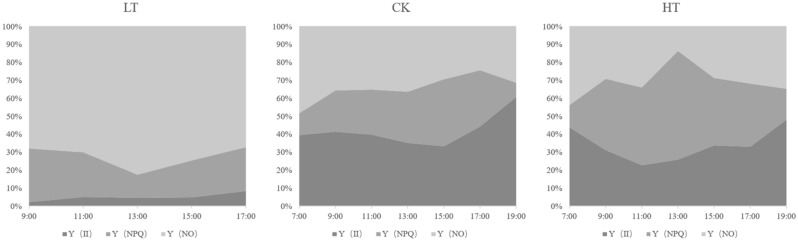
Light energy utilizations under different temperatures. Y (II): energy used for photosynthesis. Y(NO): PSII nonregulatory energy dissipation. Y (NPQ): PSII regulatory energy dissipation. The size of the areas in the graphs represents the energy proportion allocated to each part of the total absorbed energy.

The area map shows the light energy distribution for *A. mongolicus* under different temperature conditions (Fig. 2). The light energy absorbed by leaves can be divided into three categories according to their uses: photosynthesis Y(II), nonregulatory heat dissipation Y(NO) and regulatory energy dissipation Y(NPQ). The energy allocated to Y(NO) accounts for a large proportion in the LT group. Y(NO) is an important indicator of light damage, and a high proportion indicates that photochemical energy conversion and protective regulation mechanisms cannot completely consume the light energy absorbed by plants. At this time, plants may have been damaged or have not been damaged at present but will be damaged if they continue to receive light. Most of the energy absorbed by the HT group is used for Y(II) and Y(NPQ). High values of Y (NPQ) indicates that the plants accept excess light, but they can still protect themselves from damage by adjusting some processes (such as dissipating excess light energy into heat).

**Figure 3:**
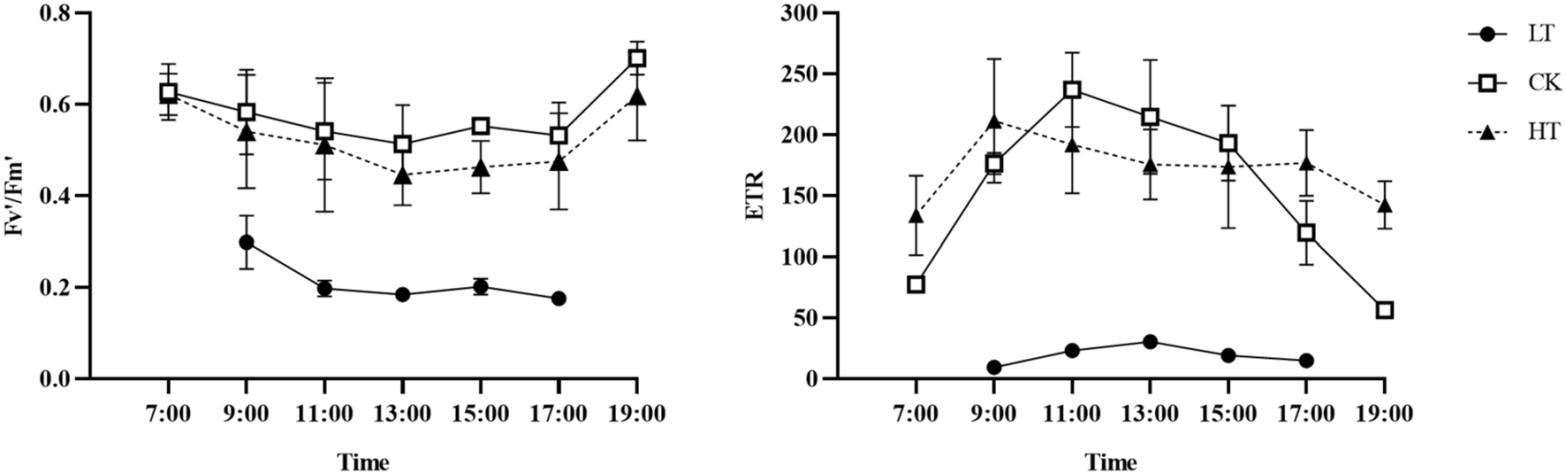
Diurnal changes in Fv'/Fm' and ETR under different temperatures. Fv'/Fm': effective photochemical quantum yield and ETR: electron transfer rate.

The effective photochemical quantum yield (Fv'/Fm') and ETR are lower in the LT group than in the HT group (Fig. 3). This shows that low temperatures significantly affect the primary electron capture efficiency and electron transfer efficiency of PSII. The maximum photochemical quantum yield Fv/Fm) of PSII can be used to characterize the photochemical efficiency of plants and indicates the primary light energy conversion efficiency of PSII as well as the potential activity of PSII. Under suitable environmental conditions, Fv/Fm is generally between 0.75 and 0.85. The Fv/Fm for the LT group is 0.32, which is far below than the normal value and indicates that the function of PS II is damaged in the subzero low-temperature environment. The Fv/Fm values of the CK and HT groups were both 0.83, which were in the normal range.

We conducted statistical analysis on the correlations of various photosynthetic parameters. The results show that A is highly correlated with intercellular CO_2_ (C_i_) and leaf temperature (T_leaf_) in the LT group, while A is highly correlated with stomatal conductance (g_sw_) and evaporation (E) in the HT group (Table 1). These results indicate that low temperatures have a great impact on the photosynthesis of *A. mongolicus.*, and the net photosynthetic rate is mainly limited by the stomata and water in the HT group.

**Table 1:** Correlations of photosynthetic parameters under different temperatures.

	A	C_i_	T_leaf_	Qin	g_sw_	E
**LT**						
A	1					
C_i_	− 0.927*	1				
T_leaf_	0.906*	− 0.820	1			
Q_in_	0.697	− 0.830	0.827	1		
g_sw_	− 0.508	0.253	− 0.739	− 0.389	1	
E	− 0.111	− 0.117	− 0.431	− 0.178	0.905*	1
**HT**						
A	1					
C_i_	− 0.219	1				
T_leaf_	− 0.604	− 0.461	1			
Q_in_	− 0.326	− 0.657	0.934**	1		
g_sw_	0.992**	− 0.125	− 0.650	− 0.384	1	
E	0.968**	− 0.383	− 0.393	− 0.105	0.949**	1

Bold indicates a significant correlation between the two indicators. *P < 0.05, **P < 0.01.

### Transcriptome results of A. mongolicus under different temperatures

**Figure 4:**
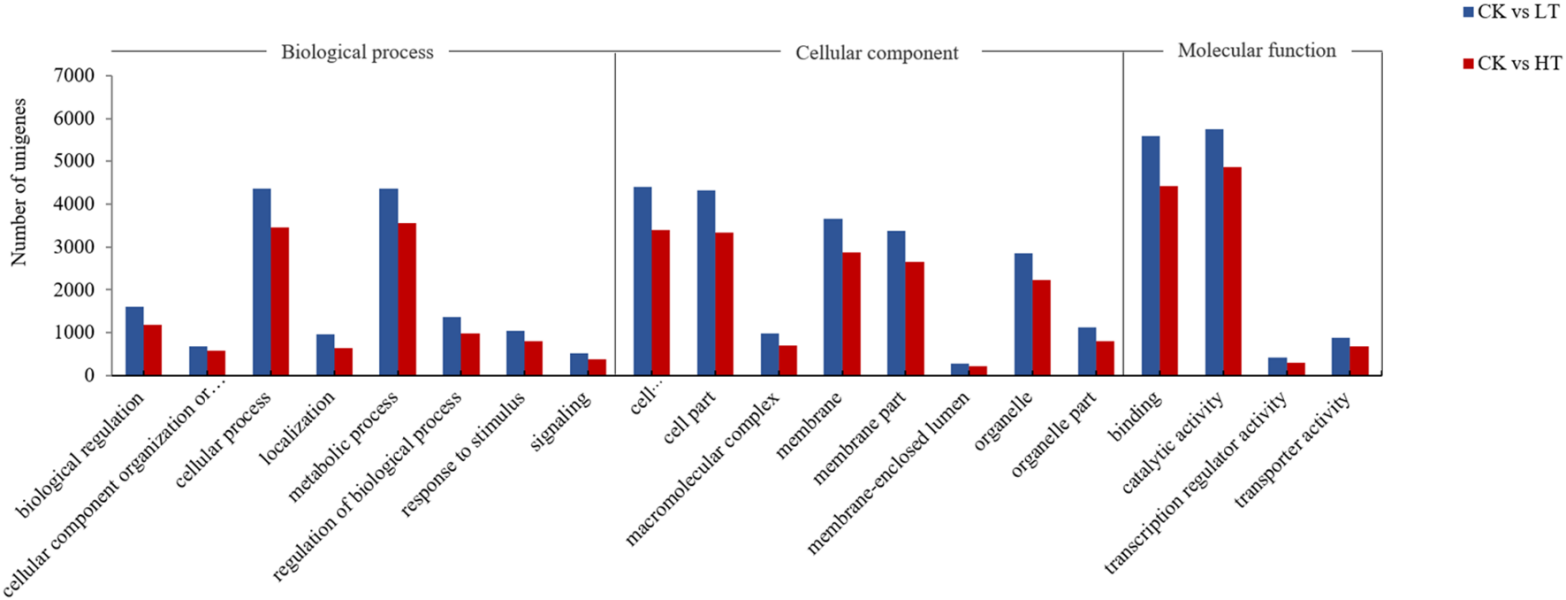
Gene Ontology (GO) functional classification analysis. The differentially expressed genes were classified into 50 categories, and the figure shows only those 20 categories with more than 200 differentially expressed genes.

The RNA-Seq sequencing results were good. The GC contents were relatively consistent, and the base content distribution of clean reads was good, with a Q30 value greater than 85%, which met the requirements for database construction (Table S1). Taking |log2FC|> 2 and the average FPKMs of samples among groups > 1 as the standard, the significantly enriched differential genes were screened out. There were 18,904 differentially expressed genes (DEGs) in the CK vs. LT groups and 15,326 DEGs in the CK vs. HT groups. The Gene Ontology (GO) project is divided into three functional categories: biological processes, cellular components and molecular functions. The results of GO classification in the CK vs. LT groups and the CK vs. HT groups were similar. In the biological processes, the numbers of DEGs in cellular processes and metabolic processes were large; in the cell component, the number of DEGs in cell and cell part was large; and in the molecular functions, the number of DEGs with binding and catalytic activity was large (Fig. 4).

**Figure 5:**
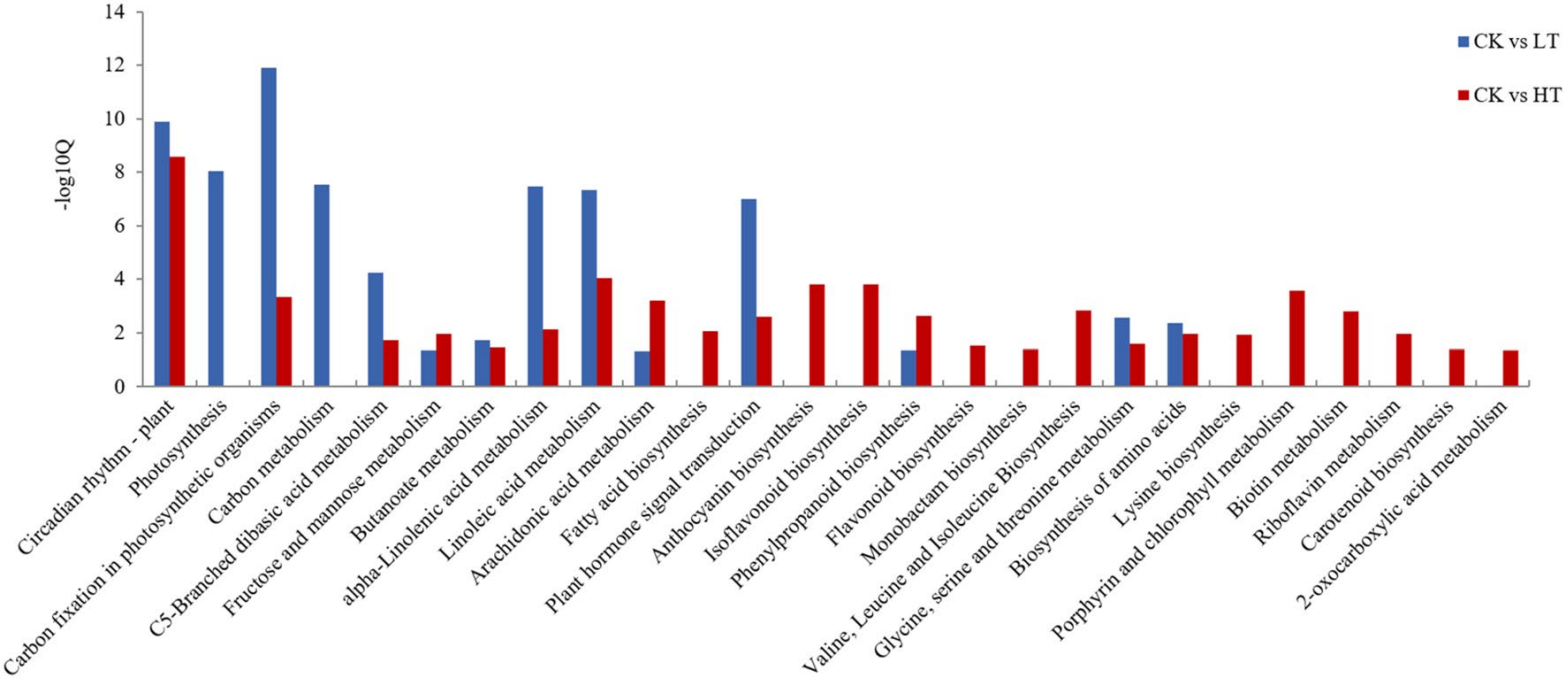
KEGG pathway enrichment map (Q value < 0.05). The Q value is obtained after P value correction, and Q value < 0.05 indicates significance.

The KEGG pathway enrichment analysis of the DEGs revealed that CK vs. LT had 15 enrichment pathways and CK vs. HT had 24 enrichment pathways (Fig. 5). Circadian rhythm, photosynthesis, lipid metabolism, carbohydrate metabolism, plant hormones and other metabolic pathways were significantly enriched in the LT group. In winter, the days are short, and the nights are long. The circadian rhythm will affect the use of light energy by *A. mongolicus.*, and low temperatures will affect the activity of biological enzymes and thus affect photosynthesis. In the HT group, metabolic pathways such as circadian rhythm, carbon fixation, carotenoid biosynthesis, secondary metabolites, cofactors and vitamin metabolism, lipid metabolism, and amino acid metabolism were significantly enriched. Studies have shown that when plants are subjected to environmental stresses such as high temperatures or strong light, their photosynthesis will be inhibited, which leads to excess amounts of light energy. Excessive light energy will cause plants to produce large amounts of active free oxygen radicals, destroy the photosynthetic apparatus, and eventually cause photooxidation and even photodamage^7^. Among them, the metabolism of carotenoids, secondary metabolites, cofactors and vitamins are all related to the removal of reactive oxygen species.

**Figure 6:**
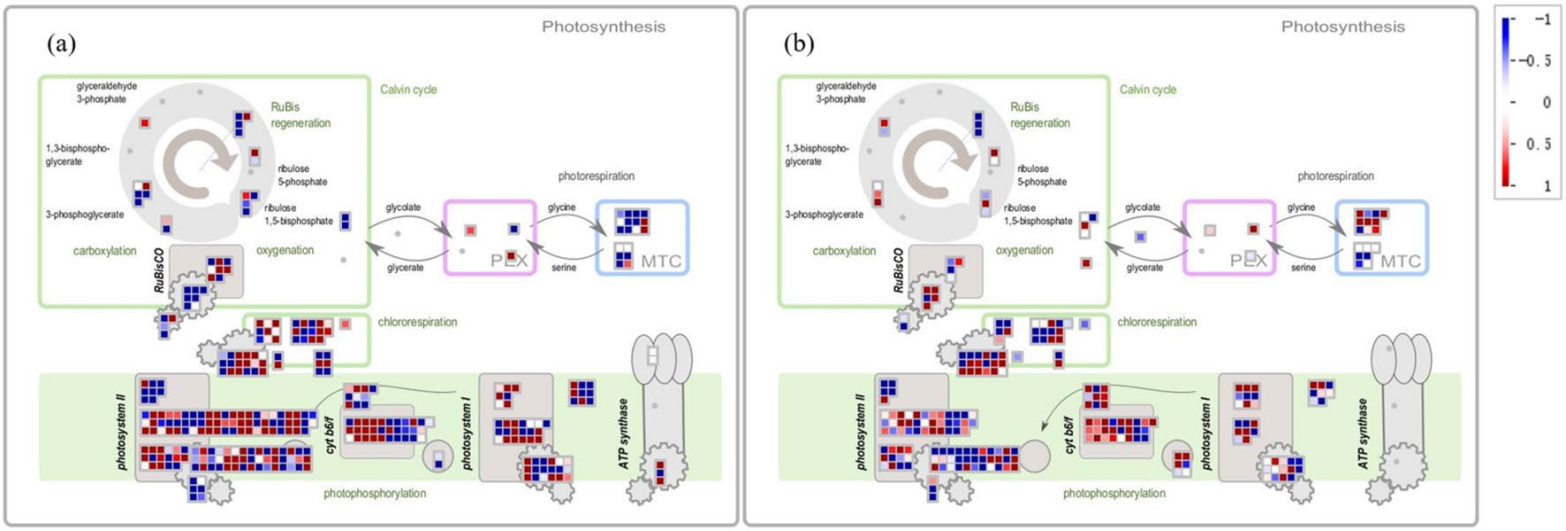
Differential gene expressions in the photosynthetic pathway under (**a**) low temperature stress and (**b**) high temperature stress. The shades of the colours indicate the levels of differential expression, and each square represents a differentially expressed gene.

Photosynthesis is an important physiological process in plants. MapMan's photosynthesis pathway includes four parts: photosynthetic phosphorylation, chloroplast respiration, the Calvin cycle, and photorespiration. Under low-temperature conditions, the difference in the LHCI complex in PSI was very significant (Fig. 6a). The responses of PSII, ATP synthase complex assembly factor (BFA3), and subcomplex A during chloroplast respiration were all significantly different. Under high-temperature conditions, the DEGs of the LHCII complex and of the assembly and maintenance of PSII were significantly changed (Fig. 6b). The DEGs assembled by Sedum heptulose-1,7-bisphosphatase (SBPase) and the Rubisco enzyme were significantly enriched in the Calvin cycle.

**Figure 7:**
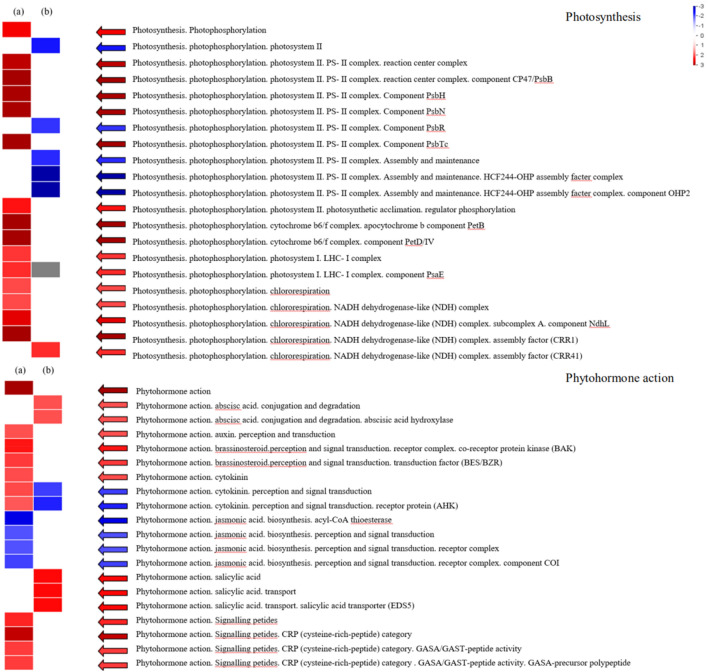
PageMan analysis of differential gene expressions in photosynthesis (above) and plant hormone action (below). (**a**) CK versus LT groups and (**b**) CK versus HT groups. The shades of the colours indicate the levels of differential expression, and each square represents a differentially expressed gene.

We used PageMan to analyse the DEGs with the Wilcoxon test. The results showed that the photosynthetic phosphorylation of photosynthesis exhibited differences under low-temperature conditions (the top part of Fig. 7). The PSII complex, PSII light response, LHCI complex, PsaE and chloroplast respiration showed differences in gene expression. High-temperature conditions mainly affect the assembly and maintenance of PSII and chloroplast respiration. The effect of phytohormones (the lower part of Fig. 7) was only significant under low-temperature conditions, and these effects on jasmonic acid (JA), auxin, brassinosteroids, cytokinins and signal peptides all changed significantly. The abscisic acid (ABA), salicylic acid and cytokinin levels in *A. mongolicus.* vary greatly in high-temperature environments.

### qRT–PCR validation

**Figure 8:**
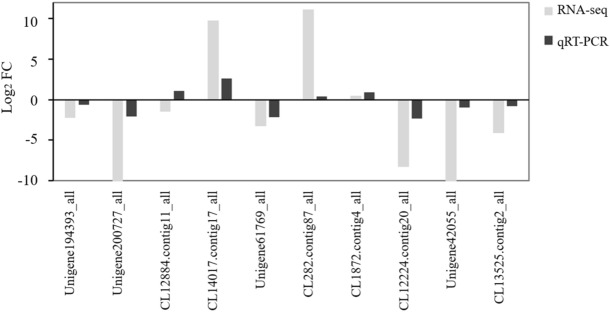
Comparisons of the qRT–PCR and RNA-seq results.

To verify the validity of the transcriptome data, 10 DEGs related to photosynthesis were randomly selected for qRT–PCR analysis, and 10 comparison groups were selected for comparisons with the transcriptional sequencing results. The results showed that the expression patterns of 9 out of 10 genes were consistent with the transcriptome sequencing results (Fig. 8), which indicated that the transcriptome sequencing results were relatively reliable in this study.

## Discussion

### DEGs under low-temperature stress

**Figure 9:**
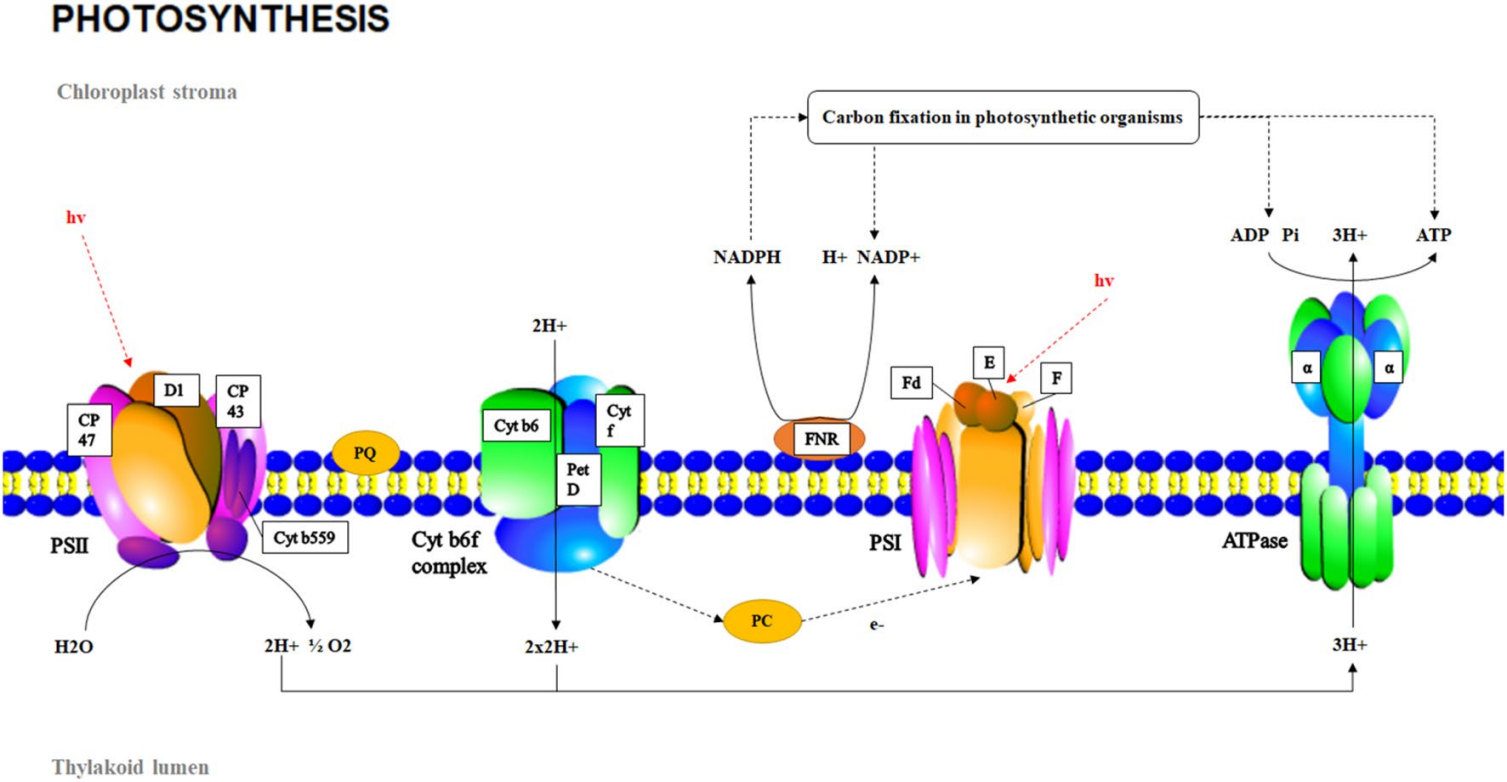
Photosynthesis of *A. mongolicus* under low-temperature stress. The areas outlined by white boxes indicate the differentially expressed genes in these structures.

The results from the field experiments indicated that the daily mean values of A, Fvʹ/Fmʹ, ETR and Fv/Fm decreased in the LT group, the PSII function was impaired, and the photosynthetic capacity was weakened. Through the specific analysis of the “Photosynthesis” pathway (pathway ID ko00195) in the LT group, it was found that PSII, the cytochrome b6f. complex (Cyt b6f.), PSI and ATPase exhibited differential gene expressions. Figure 9 shows the structural pattern diagram for photosynthesis. The parts marked by white boxes indicate that the structure has DEGs. The gene expressions of CP43, CP47, D_1_ protein and Cytb559 of PSII changed. The inner peripheral antenna pigment proteins, CP43 and CP47, of PSII bind to chlorophyll. They accept the excitation energy transferred from the surrounding antenna complex and transfer this energy to the reaction centre complex. Changes in CP43 and CP47 affect the absorption and transmission of light energy. In the PSII reaction centre, light energy is converted into chemical energy. P680 absorbs light and is excited to become P680*, and then transfers electrons to pheophytin (Pheo). At the same time, the PSII oxygen-evolving complex obtains electrons from water molecules, the water molecules are split and releases oxygen and protons. As one of the two core proteins that compose the reaction centre complex, the D_1_ protein combines with various cofactors that are related to the original charge separation and electron transfer. The D_1_ protein plays an important role in the process of photosynthetic electron transfer. Studies have found that low temperatures can induce allosteric inactivation of the D_1_ protein, which results in changes in the structure of thylakoid membranes and hinders electron transfer^8^. As part of the reaction centre, Cytb_559_ can adjust the photoinhibition sensitivity of PSII through redox changes so that the PSII reaction centre is protected from damage^9^. The light energy absorption, energy conversion and electron transfer functions of PSII are impaired, which result in significant decreases in Fv/Fm to levels far below the normal value. The results of Xiangchun Song are similar to those presented in this paper: the PS II reaction centre of *A. mongolicus* seedlings is irreversibly inactivated or the thylakoid membrane is damaged under subzero low temperature stress, which may produce serious photoinhibition. However, Song believes that the peripheral antenna component of the optical system is more affected than the core complex at low temperatures, which was not observed in the corresponding results in this study^10^.

The gene expressions of Cyt b_6_, PrtD and Cyt f in Cyt b6f. changed. Cyt b6f. changes not only affect the electron transport function of photosynthesis but also affect ATP synthesis. Pheo transfers the received electrons to plastid quinone (PQ). PQ receives electrons and protons to form plastid hydroquinone (PQH_2_). Then, the electrons of PQH_2_ are transferred to plastid cyanin (PC) on PSI through Cyt b6f., and hydrogen protons are released into the cavity of the thylakoid to form a transmembrane proton gradient. The transmembrane proton gradient is the driving force for ATP synthesis.

The function of PSI is to transfer electrons from PC to ferredoxin for the reduction of NADP^+^. Recent studies have found that PSI is more sensitive to light and more prone to selective photoinhibition than PS II under low temperature and weak light conditions^11,12^. The KEGG analysis results indicated that the LHCI complex, PsaF and PsaE subunits of PSI showed differential gene expressions. The main function of the LHCI light-harvesting pigment protein complex is to capture light energy. PsaF is a low-molecular-weight protein that is distributed in the membrane. Some studies have suggested that the N-terminal amino acid sequence of eukaryotic PsaF is involved in the binding of PSI and PC^13^. PsaE, PsaD and PsaC together form the docking site of ferredoxin on the PSI receptor side^14,15^. Ferredoxin and ferredoxin-NADP^+^ reductase in the photosynthetic electron transport chain are also affected, which results in hindrance of NADPH synthesis. The F-type H^+^/Na^+^ transport ATPase subunits also show differential gene expressions, which lead to impaired ATP synthesis. Low temperatures affect the ability to absorb light energy, transfer electrons, convert light energy into electric energy, and synthesize NADPH as well as ATP, which ultimately lead to declines in Fv'/Fm' and ETR and impair the photosynthesis capacity of *A. mongolicus.*

Compared with the light reaction, low temperatures have a greater impact on the dark reaction. Because the dark reaction process is composed of many complex enzymatic reactions, the enzyme activity is very susceptible to temperature. The KEGG results show that 13 related enzymes were differentially expressed in the "carbon sequestration of photosynthesis" (ko00710). The Rubisco enzyme is a key enzyme that determines the direction and efficiency of photosynthetic carbon metabolism in C3 plants and is sensitive to temperature^16^. The results also show that the expression levels of 10 differentially expressed genes of Rubisco enzymes all declined. In the Calvin cycle, the gene expressions of only transketolase and glyceraldehyde-3-phosphate dehydrogenase are not sensitive to temperature. In addition, the reduction phase of the dark reaction requires the use of NADPH and ATP that are produced by the light reaction. The inhibition of NADPH and ATP synthesis will inevitably affect the normal progression of the Calvin cycle.

Chloroplast respiration is an O_2_-dependent electron transport pathway in chloroplasts. Chloroplast respiration includes the nonphotochemical reduction of PQ by NAD(P) H and the reoxidation of PQ by terminal oxidase, which can consume excess electrons to protect plants from damage due to photooxidation.

**Figure 10:**
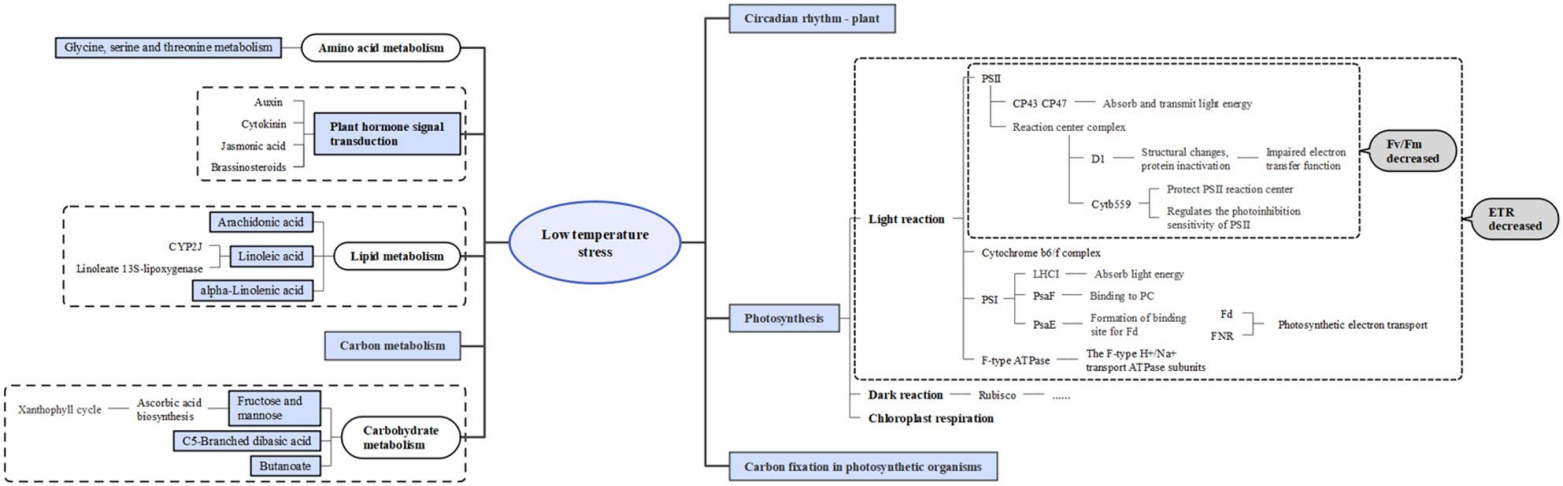
The regulatory mechanism of *A. mongolicus* under low-temperature stress. The white ovals represent the enriched metabolic pathways. The blue rectangles represent significantly enriched KEGG metabolic pathways. The pathways are followed by the physiological structures and substances or physiological processes in which the expressions of related genes change.

Figure 10 shows the partial KEGG enrichment metabolic pathway in the LT group. There were three significant enrichment pathways related to carbohydrate metabolism: fructose and mannose metabolism (ko00051), butanoate metabolism (ko00650) and C5-branched dibasic acid metabolism (ko00660). The metabolism of fructose and mannose includes the ascorbic acid biosynthetic pathway. Ascorbic acid (ASA), also known as vitamin C, can be used as a cofactor of violaxanthin de-epoxidase to participate in the lutein cycle and consume excess light energy and protect plants from harm.

Low temperatures damage cell membranes first. Increasing the mass fraction of unsaturated fatty acids in the membrane is beneficial to improve the stability and fluidity of the membrane. Some studies have shown that the degree of unsaturation of fatty acids in adult leaves of *A. mongolicus* that grow naturally in the field is lower in summer and higher in autumn and winter^17^. The significantly enriched pathways related to unsaturated fatty acid metabolism were alpha-linolenic acid metabolism (ko00592), linoleic acid metabolism (ko00591) and arachidonic acid metabolism (ko00590). Various proteins, such as linoleate 13S-lipoxygenase and cytochrome P_450_ family 2 subfamily J (CYP2J), which are involved in the metabolism of linoleic acid, showed differences in their gene expressions. Linoleate 13S-lipoxygenase is a common lipoxygenase in plants that can catalyse the production of precursors of several important compounds, including jasmonic acid. CYP2J is a group of P_450_ haem thiolate proteins, which are mainly distributed on the endoplasmic reticulum and inner mitochondrial membrane and are involved in the synthesis of sterol hormones, including brassinosteroids. Because light systems are distributed on the thylakoid membrane, damage to this membrane will affect the progress of plant photosynthesis.

Plant hormone signal transduction (ko04075) plays an important role in plant resistance to stress. Studies have shown that JAs have physiological functions, such as inducing stomatal closure, inhibiting photosynthesis, promoting respiration and promoting leaf senescence^18,19^. Treating plants with exogenous methyl jasmonate can induce the transcription of the heat shock protein family, increase the synthesis of antioxidants, reduce lipoxygenase activity and enhance the ability of plants to resist cold damage^20^.

**Figure 11:**
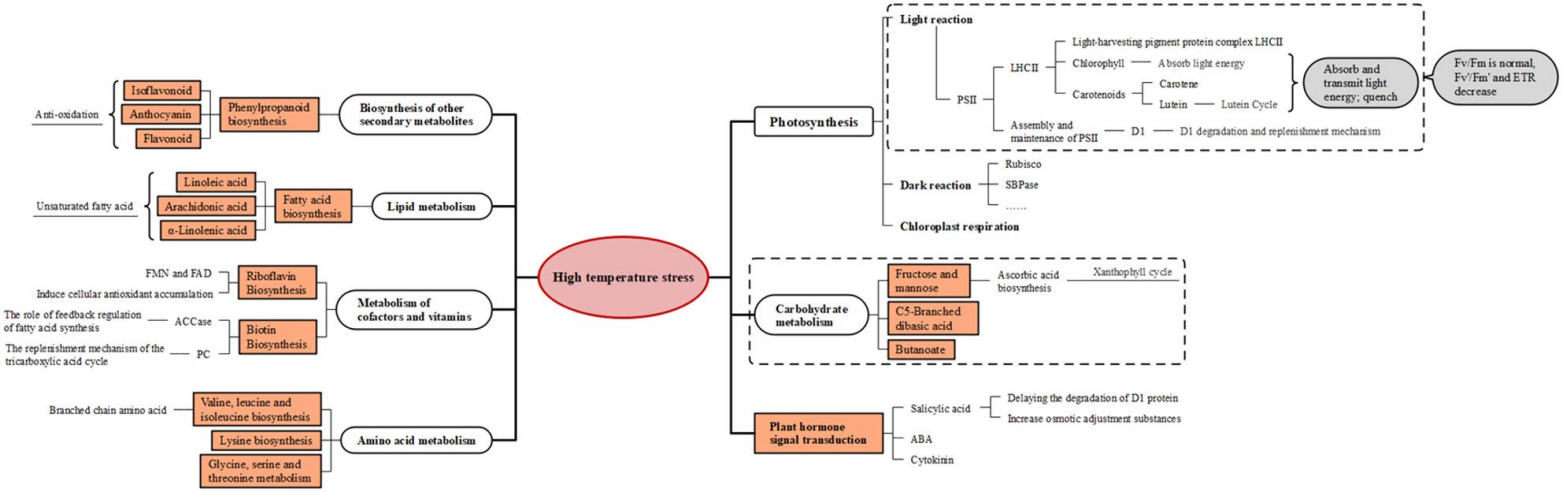
The regulatory mechanism of *A. mongolicus.* under high-temperature stress. The white ovals represent enriched metabolic pathways. The red rectangles represent significantly enriched KEGG metabolic pathways. The pathways are followed by the physiological structures and substances or physiological processes in which the expressions of related genes change.

Figure 11 shows the regulatory mechanism of *A. mongolicus* in the HL group. The MapMan analysis results show that the DEGs of the LHCII complex and those for the assembly and maintenance of PSII are significantly changed. LHCII contains chlorophyll and carotenoids, which can capture and transmit light energy. Chlorophyll is an important photosynthetic pigment that captures light energy and drives electrons to the reaction centre. The chlorophyll molecule in the reaction centre is related to photochemical quenching. The entire chlorophyll biosynthesis process (e.g., L-glutamyl-tRNA → chlorophyll a → chlorophyll b) involves 15 enzymes. The analysis found that 4/5 of the enzymes’ expression genes were changed. Carotenoids include carotene and lutein, and their synthesis is affected by high temperatures. Lutein participates in the lutein cycle, which can dissipate excess light energy and prevent membrane lipids from being peroxidized and thus maintain the stability of the thylakoid membrane structure and protect *A. mongolicus.* from high temperature stress and strong light stress.

The D_1_ protein in the PSII reaction centre is rapidly degraded under strong light conditions. To maintain the normal physiological needs of plants, the degraded D_1_ protein will be replaced by the new D_1_ protein that is produced by the repair mechanism. The reversible inactivation of the PSII reaction centre can protect the photosynthetic system and avoid destruction. This may be the reason for the significant changes in the DEGs that are involved in the assembly and maintenance of PSII.

Rubisco is the main site for high-temperature inhibition of the Calvin cycle^16^. The KEGG analysis found that there were 7 (4↑, 3↓) DEGs of Rubisco. SBPase catalyses the conversion of sedum heptulose-1,7-diphosphate (SBP) into sedum heptulose-7-phosphate (S7P) in the renewal phase. Under low-temperature stress, only transketolase and glyceraldehyde-3-phosphate dehydrogenase remained unchanged in the Calvin cycle. In addition, NDH-mediated cyclic electron transfer may decreased the photooxidation damage that is caused by high-temperature stress by shunting the excess electrons that were generated by the inhibition of CO_2_ assimilation to the chloroplast respiratory pathway^21^.

In the HT group, the net photosynthetic rates of the leaves showed two peaks on the diurnal change curves, and there was an obvious phenomenon of midday photosynthesis depression. The daily average A values were greater than those of the CK group. These results show that *A. mongolicus* has a complete photosynthetic structure protection mechanism and can adapt to high-temperature environments. The pathway of significant enrichment related to carbohydrate metabolism in the HT group was the same as that in the LT group. The enrichment degrees of the fructose and mannose metabolic pathways were higher only in the HT group, and C5-branched dibasic acid metabolism and butanoate metabolism were higher in the LT group.

Under high temperature and strong light conditions, the balance between production and removal of reactive oxygen species (ROS) in plant cells was broken, and large amounts of reactive oxygen species accumulated in the cells. Active oxygen can cause lipid peroxidation of the biomembrane, enlarge membrane pores, increase the permeability, and affect the spatial structures of enzymes on the membrane, which thus leads to chloroplast destruction. In severe cases, ROS will cause serious injury or even death to plants^22^. The gene expressions of FabH and acetyl-CoA carboxylase (ACCase) changed during the synthesis of unsaturated fatty acids in the HT group.

There are two types of active oxygen scavenging mechanisms in plants. (1) The enzymatic detoxification system: superoxide dismutase (SOD), ascorbate peroxidase (APX), and catalase (CAT). (2) Nonenzymatic antioxidants: ASA, carotenoids, glutathione, mannitol, and flavonoids^23^.

Secondary metabolites result from long-term adaptation of plants to their environments. They can improve the ability of plants to protect themselves, compete for survival, and coordinate the relationship between plants and the environment. The significant enrichment pathways related to the biosynthesis of secondary metabolites in the HT group consisted of phenylpropane biosynthesis (ko00940), flavonoid biosynthesis (ko00941) and isoflavone biosynthesis (ko00943). The phenylpropanoid biosynthesis pathway is one of the three main secondary metabolic pathways in plants. It starts from phenylalanine and generates different phenylpropane metabolites through multistep reactions, such as flavonoids, isoflavones, anthocyanins and lignin^24,25^. Anthocyanins can protect plants from light damage by quenching free oxygen radicals and reducing the absorption of light energy. Hughes studied 10 species of evergreen broad-leaved trees and found that red leaves containing anthocyanins always maintained higher Fv/Fm levels than green leaves. Fv'/Fm' is related to nonphotochemical quenching. This means that trees with red leaves rely more on the light-damage defence function of anthocyanins than on the light-damage defence mediated by lutein^26^.

Riboflavin metabolism (ko00740) and biotin metabolism (ko00780) are two significantly enriched cofactors and vitamin metabolic pathways. Riboflavin is the precursor of flavin mononucleotide (FMN) and flavin adenine dinucleotide (FAD). As a prosthetic group of flavinases, FAD participates in multiple biochemical processes, such as mitochondrial electron transport, photosynthesis, fatty acid oxidation and folate metabolism, in plants^27^. Riboflavin can induce antioxidant accumulations in plant cells and can also promote plant growth by affecting the ethylene signalling pathway^28^. Biotin (e.g., V_H_ or V_B7_), as an essential cofactor for biotin-dependent carboxylase, plays an important role in the life activities of plants. Common biotin-dependent carboxylase enzymes are pyruvate carboxylase (PC) and ACCase. PC is present in the mitochondria and participates in the replenishment mechanism of the tricarboxylic acid cycle. ACCase plays a pivotal role in the feedback regulation of fatty acid synthesis and is the site of action for the feedback regulation of fatty acid synthesis^29^.

The four pathways related to amino acid metabolism showed differences in the HT group. The enrichment degrees of each pathway were as follows: valine, leucine and isoleucine biosynthesis (ko00290) > biosynthesis of amino acids (ko01230) > lysine biosynthesis (ko00300) > glycine, serine and threonine metabolism (ko00260). The branched chain amino acids, valine, leucine and isoleucine and their derivatives, are beneficial to plant growth and plant responses to stress^30^. As an essential amino acid, lysine metabolism affects many physiological reactions, such as the tricarboxylic acid cycle, abiotic and biotic stress responses, and starch metabolism^31^. The glycine, serine and threonine metabolic pathways combined with the GO enrichment results showed that the genes related to glycine catabolism and glycine dehydrogenation/decarboxylase activity changed greatly. It is known that when the activity of mitochondrial glycine decarboxylase increases, both photorespiration and photosynthesis will increase^32^.

In terms of hormones, salicylic acid, cytokinin, and abscisic acid (ABA) can improve plant active oxygen scavenging ability. Salicylic acid can decrease the damage to seedlings due to high temperatures by improving the ability of plants to resist oxidative stress and increasing the contents of osmotic adjustment substances in cells^33^. Salicylic acid also has the function of delaying the degradation of D_1_ protein and speeding up the recovery of D_1_ protein when high temperatures are no longer present^34^. ABA can improve the heat tolerance of plants by regulating the expressions of heat stress-induced genes at the transcriptional level^35^.

In conclusion, *A. mongolicus* has weak resistance to low temperatures and good adaptation to high temperatures. At the physiological level, under low-temperature stress, the proportion of Y (NO) increased, the function of PSII was damaged, and photosynthesis was inhibited. *A. mongolica* maintains normal physiological activities by regulating the circadian rhythm, increasing the synthesis of unsaturated fatty acids and changing the effects of plant hormones. Under high-temperature stress, *A. mongolicus* maintains normal photosynthesis by adjusting g_sw_ as well as water utilization and by increasing the proportion of Y (NPQ). At the same time, *A. mongolicus* uses LHCII to consume excess energy, continuously assembles and maintains the normal function of PSII, and changes the types of antioxidants, such as by synthesizing anthocyanins, flavonoids, and isoflavones, to protect itself from injury. In addition, the porphyrin and chlorophyll metabolisms, carotenoid metabolism, plant hormones, amino acid metabolism, unsaturated fatty acid synthesis and other metabolic pathways that are related to the differentially expressed genes changed greatly.

## Materials and methods

### The research set

Mengxi town is located in the Etuoke Banner, Ordos city, western Inner Mongolia (106°43′—108°54′E, 38°18′—40°11′N), which is the area with natural distributions of *A. mongolicus* populations. The climate type is a typical temperate continental desert climate, which has the characteristics of large temperature differences between day and night, clear seasonality, cold in winter and hot in summer, little rain, and constant wind and sand. From 2010 to 2020 (2010.1.1–2020.12.31), the annual average temperature in this location was 10.3 °C, the lowest temperature was − 26.7 °C, and the highest temperature was 41.0 °C; the average annual rainfall was only 120.4 mm (meteorological data source: Reliable Prognosis; website https: https://rp5.ru/docs/about/cn).

### Experimental materials

Five individual *A. mongolicus* plants with similar sizes were selected for labelling (height: 90–100 cm, crown diameter: 100–150 cm, and base diameter: approximately 0.3 cm). All of these individuals grew in similar microenvironments. We took April as the control group (CK, monthly average temperature of 15.1 °C); January as the low-temperature treatment group (LT, monthly average temperature of − 7.5 °C); and July as the high-temperature treatment group (HT, monthly average temperature of 26.8 °C) and measured the photosynthetic physiological indices under natural conditions.

The collection of leaf samples that were required for RNA-seq analysis was carried out simultaneously with the physiological experiments. Ten to 15 healthy leaves were collected from each labelled plant, wrapped in tinfoil and quickly stored in liquid nitrogen. The leaf samples of 5 plants that were collected under different treatment conditions were mixed, and three replicates were taken for each treatment.

### Field experiment methods

During the LT (January), CK (April) and HT (July) periods, clear and cloudless weather was selected to measure the photosynthetic physiological indices and diurnal changes in chlorophyll fluorescence. Mature leaves located at the 2nd-4th positions on the tops of the labelled plant branches were selected, 5 leaves were selected from each plant, and measurements were repeated at least 5 times for each leaf. The measurement time was from 7:00 to 19:00, and measurements were conducted every two hours for a total of 7 times. Affected by the local sunrise and sunset time, the LT measurement time was from 9:00 to 17:00. The photosynthetic and fluorescence parameters were measured synchronously by an Li-6800 portable photosynthetic instrument (LI-COR, Inc., Lincoln, NE, USA) and an Li-6400xt portable photosynthetic instrument (LI-COR, Inc., Lincoln, NE, USA).

### Transcriptome experiment method

BGI Co., Ltd. (Beijing) completed the RNA extractions, library construction and sequencing of leaf slices of *A. mongolicus*. An ethanol precipitation protocol and CTAB-PBIOZOL reagent were used for purifying the total RNA from plant tissues. Subsequently, the total RNA was qualified and quantified using a Nano Drop and Agilent 2100 bioanalyzer (Thermo Fisher Scientific, MA, USA).

The mRNA library type used was the BGISEQ-500 transcriptome (BGIShenzhen, China). Trinity^36^ (v2.0.6) was used to de novo assemble the clean reads (i.e., remove PCR duplications to improve the assembly efficiency) and the assembled transcripts were then clustered with Tgicl (v2.1) to remove redundancy to obtain unigenes. The quality of the assembled transcripts was evaluated by using the single copy direct homology database, BUSCO. Bowtie2 (v2.2.5) was used to align the clean reads to the resequenced transcriptome sequence, and RSEM^37,38^ (v1.2.8) was used to calculate the gene expression levels of each sample. Through DEGseq2 (v1.4.5)^39^ analysis, the genes with difference multiples of more than 2 times and Q-values ≤ 0.001 were screened as significantly differentially expressed genes. Then, GO (http://www.geneontology.org/) and KEGG (https://www.kegg.jp/) enrichment analysis, MapMan (v3.6.0) analysis and PageMan (v3.5.0) analysis were carried out.

To verify the reliability of the transcriptome results, 10 genes related to photosynthesis were randomly selected for qRT-PCR verification. Actin was used as the internal reference gene, and the internal reference in this study was UniGene 75062_ All. See Table S3 for the primer design. The specific experimental process consisted of RNA extraction and quality inspection-reverse transcription-fluorescence quantitative PCR amplification-data output analysis. The primers were designed by using Primer Express Software (v2.0) from the ABI company. The reactions were carried out in 384-well plates on an ABI ViiA 7 PCR instrument, and each sample was subjected to three parallel experiments. The relative expressions of the target genes were calculated by using the 2-△△Ct method.

### Statement

We declare that we strictly abide by the guidelines of the IUCN Policy Statement on Research Involving Species at Risk of Extinction when collecting and using wild *A. mongolicus* leaves in Ordos, Inner Mongolia, China. We used non-lethal sampling methods to collected 5–10 leaves from each healthy plant. The amount of leaf specimens we collected does not increase the extinction risk of *A. mongolicus*, and the research presented is critical to assist in the conservation of this species.

We have obtained the collection permit from the Administration of West Ordos National Nature Reserve in Ordos City. The collected leaf samples of *A. mongolicus* were stored in the Herbarium of the School of Life and Environmental Sciences, Minzu University of China (China) (temporarily placed in Laboratory 1103).

Species identifier: Liu Bo, Minzu University of China.

## Supplementary Information

Supplementary Tables.

## Data Availability

The data underlying this article are available in NCBI, and can be accessed with https://dataview.ncbi.nlm.nih.gov/object/PRJNA784165?reviewer=dh78oa7773uc8ua7d69l97skqf. Correspondence and requests for materials should be addressed to S.S.
