# Supplementary material for: Physiological and transcriptome analyses reveal the response of Ammopiptanthus mongolicus to extreme seasonal temperatures in a cold plateau desert ecosystem
Source: Sci Rep. 2022 Jun 23;12:10630. doi: 10.1038/s41598-022-14402-8 (PMC9226188; doi:10.1038/s41598-022-14402-8)
Supplement: Supplementary file 1 — Supplementary Tables. [file 41598_2022_14402_MOESM1_ESM.pdf]

Table S1. Results of sequencing data quality statistics

| Sample | Total Raw Reads (M) | Total Clean Reads (M) | Total Clean Bases(Gb) | Clean Reads Q20(%) | Clean Reads Q30(%) | Clean Reads Ratio(%) | （GC%） |
|---|---|---|---|---|---|---|---|
| LT_M1 | 47.44 | 42.85 | 6.43 | 96.69 | 88.23 | 90.34 | 40.35 |
| LT_M2 | 49.19 | 44.78 | 6.72 | 96.62 | 88 | 91.03 | 40.24 |
| LT_M3 | 49.19 | 44.04 | 6.61 | 96.62 | 88 | 89.52 | 40.21 |
| CK_M1 | 49.08 | 42.85 | 6.43 | 96.52 | 87.94 | 87.3 | 40.44 |
| CK_M2 | 50.83 | 44.19 | 6.63 | 96.35 | 87.46 | 86.94 | 40.39 |
| CK_M3 | 50.83 | 44.61 | 6.69 | 96.48 | 87.84 | 87.77 | 40.25 |
| HT_M1 | 50.83 | 44.58 | 6.69 | 96.37 | 87.53 | 87.7 | 40.32 |
| HT_M2 | 50.83 | 44.57 | 6.69 | 96.48 | 87.84 | 87.69 | 40.27 |
| HT_M3 | 49.08 | 43.63 | 6.54 | 96.25 | 87.22 | 88.9 | 40.38 |

Table S2. The first 20 GO terms of differential expressed genes in CK vs HT

| GO Term ID | GO Term | Level 1 | Level 2 | Gene Num | Q value |
|---|---|---|---|---|---|
| GO:0010411 | xyloglucan metabolic process | biological_process | cellular process | 38 | 2.88E-09 |
| GO:0016762 | xyloglucan:xyloglucosyl transferase activity | molecular_function | catalytic activity | 37 | 2.88E-09 |
| GO:0042546 | cell wall biogenesis | biological_process | cellular component organization or biogenesis | 37 | 7.44E-09 |
| GO:0006546 | glycine catabolic process | biological_process | biological regulation | 22 | 4.14E-08 |
| GO:0016747 | transferase activity, transferring acyl groups other than amino-acyl groups | molecular_function | catalytic activity | 74 | 8.45E-08 |
| GO:0005618 | cell wall | cellular_component | cell | 84 | 1.05E-06 |
| GO:0004857 | enzyme inhibitor activity | molecular_function | molecular function regulator | 23 | 3.41E-06 |
| GO:0004375 | Glycine dehydrogenase (decarboxylating) activity | molecular_function | catalytic activity | 16 | 1.08E-05 |
| GO:0048046 | apoplast | cellular_component | extracellular region | 46 | 1.66E-05 |
| GO:0005315 | inorganic phosphate transmembrane transporter activity | molecular_function | transporter activity | 27 | 9.27E-05 |
| GO:0005980 | glycogen catabolic process | biological_process | cellular process | 18 | 9.27E-05 |
| GO:0009099 | valine biosynthetic process | biological_process | cellular process | 15 | 9.27E-05 |
| GO:0004553 | hydrolase activity, hydrolyzing O-glycosyl compounds | molecular_function | catalytic activity | 115 | 0.00013 |
| GO:0005215 | transporter activity | molecular_function | transporter activity | 128 | 0.00013 |
| GO:0004657 | proline dehydrogenase activity | molecular_function | catalytic activity | 12 | 0.000147 |
| GO:0015996 | chlorophyll catabolic process | biological_process | cellular process | 16 | 0.000147 |
| GO:0042545 | cell wall modification | biological_process | cellular component organization or biogenesis | 17 | 0.000147 |
| GO:0045330 | aspartyl esterase activity | molecular_function | catalytic activity | 17 | 0.000167 |
| GO:0031408 | oxylipin biosynthetic process | biological_process | cellular process | 21 | 0.000187 |
| GO:0009097 | isoleucine biosynthetic process | biological_process | cellular process | 15 | 0.000216 |

Table S3. Primers used for qRT-PCR

| Gene ID | Forward Primer（5’→3’） | Reverse Primer（5’→3’） |
|---|---|---|
| Unigene194393_all | TGGCATCTTCAGTCATGGCT | TGGCCACAACTCTGAAGGAA |
| Unigene200727_all | GTGGCTCATTTCGTACCCGA | ACTTCCCCCCCAGGACCTAC |
| CL12884.contig11_all | CAAAGTGGAACCGGAGCAAA | TGGAGTGACCGTGACAACGA |
| CL14017.contig17_all | TTTGAAATCGGATGGCGTTT | CATGCCAAATGTGTCCGAAA |
| Unigene61769_all | CGCCAGAGTTTCAGACCCAA | GGCCTGTAGTGTTGCCCAGA |
| CL282.contig87_all | TGATCCTGCACGTATTTCGG | AGATGCGGTCAATACAGCCA |
| CL1872.contig4_all | CCTGCCACCACTCTCCATTG | CAAAGCCATGCTCCAACTCG |
| CL12224.contig20_all | GGAGCCCCCTTTCTGTCAAG | GCATTCCGATCTGCATTTGG |
| Unigene42055_All | CGCCTGGAGCTCGTATGAGA | GCTTGGACGAAGCGGAAGAT |
| CL13525.contig2_all | ATGGCATCAGCTGCTTCAGG | TTGCCCTCACGACAAGCCTA |
| Unigene75062_All | GTCAAGGCTGGTTTTGCTGG | TCCTTTTGTCCCATGCCAAC |
